# 4-Dimethyl­amino-*N*′-(3-pyridyl­methyl­idene)benzohydrazide

**DOI:** 10.1107/S1600536810037670

**Published:** 2010-09-25

**Authors:** Yan-Wei Ding, Li-Li Ni

**Affiliations:** a College of Chemistry and Chemical Engineering, Liaoning Normal University, Dalian 116029, People’s Republic of China; b The Miyun High School Attached to Capital Normal University, Beijing 101500, People’s Republic of China

## Abstract

The title compound, C_15_H_16_N_4_O, was prepared by the reaction of pyridine-3-carbaldehyde with 4-dimethyl­amino­benzo­hydrazide in methanol. The dihedral angle between the pyridine and the benzene rings is 5.1 (3)°. In the crystal structure, the hydrazone mol­ecules are linked through inter­molecular N—H⋯O hydrogen bonds, forming chains along the *b* axis.

## Related literature

For the synthesis and biological applications of hydrazone compounds, see: Alvarez *et al.* (2008 ▶); Angelusiu *et al.* (2010 ▶); Ajani *et al.* (2010 ▶); El-Dissouky *et al.* (2010 ▶); Avaji *et al.* (2009 ▶); Fouda *et al.* (2008 ▶). For the crystal structures of similar hydrazone compounds, see: Wen *et al.* (2009 ▶); Fun *et al.* (2008 ▶); Ji & Lu (2010 ▶); Ahmad *et al.* (2010 ▶); Cui *et al.* (2009 ▶).
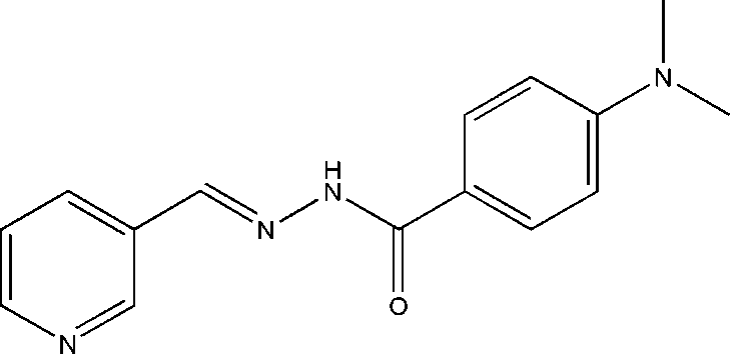


## Experimental

### 

#### Crystal data

C_15_H_16_N_4_O
*M* *_r_* = 268.32
Orthorhombic, 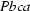

*a* = 11.513 (2) Å
*b* = 7.898 (2) Å
*c* = 30.359 (3) Å
*V* = 2760.5 (9) Å^3^
*Z* = 8
Mo *K*α radiation
μ = 0.09 mm^−1^
*T* = 298 K
0.10 × 0.07 × 0.05 mm

#### Data collection

Bruker SMART CCD area-detector diffractometer
Absorption correction: multi-scan (*SADABS*; Bruker, 2001 ▶) *T*_min_ = 0.992, *T*_max_ = 0.996
20776 measured reflections
2991 independent reflections
1163 reflections with *I* > 2σ(*I*)
*R*_int_ = 0.190

#### Refinement

*R*[*F* ^2^ > 2σ(*F* ^2^)] = 0.058
*wR*(*F* ^2^) = 0.151
*S* = 0.79
2991 reflections
186 parameters
1 restraint
H atoms treated by a mixture of independent and constrained refinement
Δρ_max_ = 0.20 e Å^−3^
Δρ_min_ = −0.25 e Å^−3^

### 

Data collection: *SMART* (Bruker, 2007 ▶); cell refinement: *SAINT* (Bruker, 2007 ▶); data reduction: *SAINT*; program(s) used to solve structure: *SHELXTL* (Sheldrick, 2008 ▶); program(s) used to refine structure: *SHELXTL*; molecular graphics: *SHELXTL*; software used to prepare material for publication: *SHELXTL*.

## Supplementary Material

Crystal structure: contains datablocks global, I. DOI: 10.1107/S1600536810037670/rz2491sup1.cif

Structure factors: contains datablocks I. DOI: 10.1107/S1600536810037670/rz2491Isup2.hkl

Additional supplementary materials:  crystallographic information; 3D view; checkCIF report

## Figures and Tables

**Table 1:** Hydrogen-bond geometry (Å, °)

*D*—H⋯*A*	*D*—H	H⋯*A*	*D*⋯*A*	*D*—H⋯*A*
N3—H3⋯O1^i^	0.89 (1)	2.16 (1)	3.035 (3)	166 (3)

Symmetry code: (i) 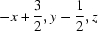.

**Fig. 1.**
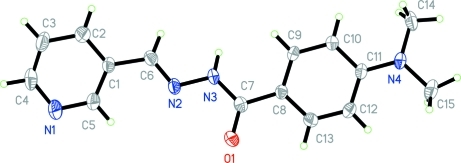
Molecular structure of the title compound with 30% probability displacement ellipsoids.

**Fig. 2.**
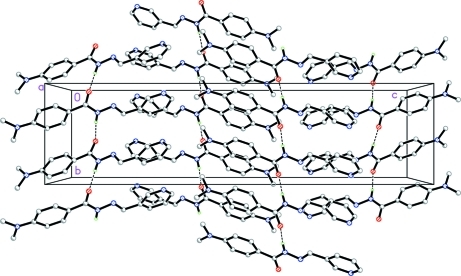
Molecular packing of the title compound, viewed along the a axis. Hydrogen bonds are shown as dashed lines.

## supplementary crystallographic information

### Comment

In the last few years, considerable attention has focused on the preparation and biological application of hydrazone compounds (Alvarez *et al.*, 2008; Angelusiu *et al.*, 2010; Ajani *et al.*, 2010; El-Dissouky *et al.*, 2010; Avaji *et al.*, 2009; Fouda *et al.*, 2008). In this paper, the crystal structure of the title new hydrazone compound is reported.

The molecular structure of the title compound is shown in Fig. 1. The dihedral angle between the pyridine and the benzene rings is 5.1 (3)°. The torsion angles C1—C6—N2—N3, C6—N2—N3—C7, N2—N3—C7—C8, and N2—N3—C7—O1 are 2.4 (3), 2.4 (3), 3.4 (3), and 0.9 (3)°, respectively. All the bond lengths are within normal values and are comparable with the similar hydrazone compounds (Wen *et al.*, 2009; Fun *et al.*, 2008; Ji & Lu, 2010; Ahmad *et al.*, 2010; Cui *et al.*, 2009). In the crystal structure, the hydrazone molecules are linked through intermolecular hydrogen bonds of type N—H···O (Table 1), forming chains along the *b* axis, as shown in Fig. 2.

### Experimental

The title compound was prepared by the reaction of pyridine-3-carbaldehyde (0.107 g, 1 mmol) with 4-dimethylaminobenzohydrazide (0.179 g, 1 mmol) in methanol at ambient temperature. Colourless block-like single crytals were formed by slow evaporation of the solution in air.

### Refinement

Atom H3 attached to N3 was located in a difference Fourier map and refined with the N3—H3 distance restrained to 0.90 (1) Å and an isotropic displacement parameter fixed at 0.08 Å^2^. All other H atoms were positioned geometrically and refined using a riding-model approximation, with C—H = 0.93–0.96 Å, and with *U*_iso_(H) = 1.2*U*_eq_(C) or 1.5*U*_eq_(C) for methyl H atoms. Crystals were small and very weakly diffracting and this is reflected in the large value of *R*_int_ (0.19), and the low ratio of observed/unique reflections (39%).

### Crystal data

**Table:**

C_15_H_16_N_4_O	*F*(000) = 1136
*M**_r_* = 268.32	*D*_x_ = 1.291 Mg m^−^^3^
Orthorhombic, *P**b**c**a*	Mo *K*α radiation, λ = 0.71073 Å
Hall symbol: -P 2ac 2ab	Cell parameters from 1003 reflections
*a* = 11.513 (2) Å	θ = 2.3–24.0°
*b* = 7.898 (2) Å	µ = 0.09 mm^−^^1^
*c* = 30.359 (3) Å	*T* = 298 K
*V* = 2760.5 (9) Å^3^	Block, colourless
*Z* = 8	0.10 × 0.07 × 0.05 mm

### Data collection

**Table:**

Bruker SMART CCD area-detector diffractometer	2991 independent reflections
Radiation source: fine-focus sealed tube	1163 reflections with *I* > 2σ(*I*)
graphite	*R*_int_ = 0.190
ω scans	θ_max_ = 27.0°, θ_min_ = 1.3°
Absorption correction: multi-scan (*SADABS*; Bruker, 2001)	*h* = −14→14
*T*_min_ = 0.992, *T*_max_ = 0.996	*k* = −10→9
20776 measured reflections	*l* = −38→37

### Refinement

**Table:**

Refinement on *F*^2^	Primary atom site location: structure-invariant direct methods
Least-squares matrix: full	Secondary atom site location: difference Fourier map
*R*[*F*^2^ > 2σ(*F*^2^)] = 0.058	Hydrogen site location: inferred from neighbouring sites
*wR*(*F*^2^) = 0.151	H atoms treated by a mixture of independent and constrained refinement
*S* = 0.79	*w* = 1/[σ^2^(*F*_o_^2^) + (0.0518*P*)^2^] where *P* = (*F*_o_^2^ + 2*F*_c_^2^)/3
2991 reflections	(Δ/σ)_max_ < 0.001
186 parameters	Δρ_max_ = 0.20 e Å^−^^3^
1 restraint	Δρ_min_ = −0.25 e Å^−^^3^

### Special details

**Table:**

Geometry. All e.s.d.'s (except the e.s.d. in the dihedral angle between two l.s. planes) are estimated using the full covariance matrix. The cell e.s.d.'s are taken into account individually in the estimation of e.s.d.'s in distances, angles and torsion angles; correlations between e.s.d.'s in cell parameters are only used when they are defined by crystal symmetry. An approximate (isotropic) treatment of cell e.s.d.'s is used for estimating e.s.d.'s involving l.s. planes.
Refinement. Refinement of *F*^2^ against ALL reflections. The weighted *R*-factor *wR* and goodness of fit *S* are based on *F*^2^, conventional *R*-factors *R* are based on *F*, with *F* set to zero for negative *F*^2^. The threshold expression of *F*^2^ > σ(*F*^2^) is used only for calculating *R*-factors(gt) *etc*. and is not relevant to the choice of reflections for refinement. *R*-factors based on *F*^2^ are statistically about twice as large as those based on *F*, and *R*- factors based on ALL data will be even larger.

### Fractional atomic coordinates and isotropic or equivalent isotropic displacement parameters (Å^2^)

**Table:**

	*x*	*y*	*z*	*U*_iso_*/*U*_eq_	
N1	0.7015 (2)	0.4212 (3)	0.79621 (8)	0.0715 (8)	
N2	0.74617 (19)	0.2877 (3)	0.66360 (7)	0.0442 (6)	
N3	0.7654 (2)	0.2199 (3)	0.62206 (7)	0.0462 (6)	
N4	0.8832 (2)	0.0665 (3)	0.41884 (7)	0.0548 (7)	
O1	0.89752 (16)	0.4234 (2)	0.60695 (5)	0.0494 (5)	
C1	0.6436 (2)	0.2668 (4)	0.73145 (8)	0.0425 (7)	
C2	0.5449 (3)	0.2126 (4)	0.75284 (10)	0.0636 (9)	
H2	0.4923	0.1416	0.7387	0.076*	
C3	0.5253 (3)	0.2652 (4)	0.79551 (10)	0.0677 (10)	
H3A	0.4595	0.2296	0.8107	0.081*	
C4	0.6033 (3)	0.3691 (4)	0.81491 (10)	0.0664 (10)	
H4	0.5871	0.4069	0.8433	0.080*	
C5	0.7181 (3)	0.3692 (4)	0.75487 (9)	0.0581 (9)	
H5	0.7854	0.4050	0.7407	0.070*	
C6	0.6688 (2)	0.2116 (4)	0.68628 (9)	0.0459 (8)	
H6	0.6283	0.1210	0.6742	0.055*	
C7	0.8431 (2)	0.2965 (4)	0.59507 (9)	0.0405 (7)	
C8	0.8553 (2)	0.2255 (3)	0.55060 (8)	0.0374 (7)	
C9	0.7769 (2)	0.1128 (3)	0.53203 (8)	0.0416 (7)	
H9	0.7157	0.0732	0.5491	0.050*	
C10	0.7866 (2)	0.0575 (3)	0.48906 (8)	0.0456 (7)	
H10	0.7325	−0.0185	0.4778	0.055*	
C11	0.8776 (2)	0.1154 (3)	0.46229 (9)	0.0423 (7)	
C12	0.9577 (2)	0.2268 (4)	0.48112 (9)	0.0506 (8)	
H12	1.0198	0.2656	0.4643	0.061*	
C13	0.9464 (2)	0.2797 (3)	0.52384 (9)	0.0470 (8)	
H13	1.0012	0.3540	0.5354	0.056*	
C14	0.8071 (3)	−0.0613 (4)	0.40150 (9)	0.0736 (10)	
H14A	0.8202	−0.1660	0.4168	0.110*	
H14B	0.8225	−0.0765	0.3707	0.110*	
H14C	0.7279	−0.0266	0.4055	0.110*	
C15	0.9728 (3)	0.1326 (4)	0.38994 (9)	0.0780 (11)	
H15A	0.9755	0.2537	0.3924	0.117*	
H15B	0.9556	0.1018	0.3601	0.117*	
H15C	1.0467	0.0859	0.3982	0.117*	
H3	0.728 (2)	0.124 (2)	0.6156 (9)	0.080*	

### Atomic displacement parameters (Å^2^)

**Table:**

	*U*^11^	*U*^22^	*U*^33^	*U*^12^	*U*^13^	*U*^23^
N1	0.084 (2)	0.094 (2)	0.0365 (16)	−0.0072 (18)	0.0065 (15)	−0.0122 (15)
N2	0.0460 (15)	0.0567 (17)	0.0298 (13)	0.0026 (13)	0.0005 (11)	−0.0049 (12)
N3	0.0557 (17)	0.0556 (18)	0.0274 (12)	−0.0044 (13)	0.0040 (11)	−0.0085 (12)
N4	0.0619 (17)	0.0693 (18)	0.0331 (14)	−0.0091 (15)	0.0156 (13)	−0.0065 (13)
O1	0.0555 (13)	0.0565 (13)	0.0363 (12)	−0.0069 (11)	−0.0072 (9)	−0.0053 (10)
C1	0.0428 (17)	0.0533 (19)	0.0313 (16)	0.0044 (15)	0.0021 (13)	0.0008 (14)
C2	0.050 (2)	0.087 (3)	0.054 (2)	−0.0078 (18)	0.0038 (16)	−0.0122 (18)
C3	0.058 (2)	0.103 (3)	0.042 (2)	0.004 (2)	0.0161 (17)	−0.0005 (19)
C4	0.078 (3)	0.088 (3)	0.0328 (18)	0.018 (2)	0.0087 (19)	−0.0037 (18)
C5	0.062 (2)	0.076 (2)	0.0366 (18)	−0.0078 (18)	0.0079 (16)	−0.0041 (16)
C6	0.0441 (18)	0.055 (2)	0.0382 (17)	0.0024 (15)	−0.0041 (14)	−0.0068 (15)
C7	0.0432 (18)	0.0435 (19)	0.0348 (17)	0.0083 (15)	−0.0065 (14)	0.0014 (15)
C8	0.0382 (16)	0.0434 (18)	0.0305 (16)	0.0019 (14)	−0.0009 (12)	0.0024 (13)
C9	0.0453 (18)	0.0491 (19)	0.0304 (15)	−0.0046 (15)	0.0082 (13)	0.0051 (13)
C10	0.0497 (18)	0.053 (2)	0.0340 (16)	−0.0088 (15)	0.0014 (14)	−0.0004 (14)
C11	0.0454 (18)	0.0516 (19)	0.0301 (16)	0.0027 (15)	0.0065 (14)	0.0013 (14)
C12	0.0433 (18)	0.064 (2)	0.0445 (18)	−0.0057 (16)	0.0133 (14)	−0.0009 (16)
C13	0.0407 (18)	0.057 (2)	0.0431 (18)	−0.0047 (15)	−0.0024 (14)	−0.0050 (15)
C14	0.102 (3)	0.087 (3)	0.0322 (18)	−0.014 (2)	0.0041 (18)	−0.0140 (17)
C15	0.088 (3)	0.098 (3)	0.047 (2)	−0.010 (2)	0.0301 (19)	−0.0022 (18)

### Geometric parameters (Å, °)

**Table:**

N1—C4	1.331 (4)	C6—H6	0.9300
N1—C5	1.334 (3)	C7—C8	1.469 (3)
N2—C6	1.276 (3)	C8—C9	1.388 (3)
N2—N3	1.388 (3)	C8—C13	1.394 (3)
N3—C7	1.356 (3)	C9—C10	1.380 (3)
N3—H3	0.894 (10)	C9—H9	0.9300
N4—C11	1.376 (3)	C10—C11	1.402 (3)
N4—C14	1.436 (3)	C10—H10	0.9300
N4—C15	1.451 (3)	C11—C12	1.397 (3)
O1—C7	1.236 (3)	C12—C13	1.369 (3)
C1—C2	1.377 (4)	C12—H12	0.9300
C1—C5	1.377 (4)	C13—H13	0.9300
C1—C6	1.468 (3)	C14—H14A	0.9600
C2—C3	1.379 (4)	C14—H14B	0.9600
C2—H2	0.9300	C14—H14C	0.9600
C3—C4	1.352 (4)	C15—H15A	0.9600
C3—H3A	0.9300	C15—H15B	0.9600
C4—H4	0.9300	C15—H15C	0.9600
C5—H5	0.9300		
			
C4—N1—C5	115.3 (3)	C9—C8—C7	123.8 (2)
C6—N2—N3	114.8 (2)	C13—C8—C7	119.4 (3)
C7—N3—N2	118.8 (2)	C10—C9—C8	122.3 (2)
C7—N3—H3	124.4 (19)	C10—C9—H9	118.8
N2—N3—H3	116.7 (19)	C8—C9—H9	118.8
C11—N4—C14	121.3 (2)	C9—C10—C11	120.3 (3)
C11—N4—C15	120.8 (3)	C9—C10—H10	119.8
C14—N4—C15	117.7 (2)	C11—C10—H10	119.8
C2—C1—C5	116.9 (3)	N4—C11—C12	122.5 (2)
C2—C1—C6	120.8 (3)	N4—C11—C10	119.9 (3)
C5—C1—C6	122.3 (3)	C12—C11—C10	117.5 (2)
C1—C2—C3	119.0 (3)	C13—C12—C11	121.2 (2)
C1—C2—H2	120.5	C13—C12—H12	119.4
C3—C2—H2	120.5	C11—C12—H12	119.4
C4—C3—C2	118.9 (3)	C12—C13—C8	122.0 (3)
C4—C3—H3A	120.6	C12—C13—H13	119.0
C2—C3—H3A	120.6	C8—C13—H13	119.0
N1—C4—C3	124.5 (3)	N4—C14—H14A	109.5
N1—C4—H4	117.8	N4—C14—H14B	109.5
C3—C4—H4	117.8	H14A—C14—H14B	109.5
N1—C5—C1	125.3 (3)	N4—C14—H14C	109.5
N1—C5—H5	117.4	H14A—C14—H14C	109.5
C1—C5—H5	117.4	H14B—C14—H14C	109.5
N2—C6—C1	120.1 (3)	N4—C15—H15A	109.5
N2—C6—H6	119.9	N4—C15—H15B	109.5
C1—C6—H6	119.9	H15A—C15—H15B	109.5
O1—C7—N3	121.4 (3)	N4—C15—H15C	109.5
O1—C7—C8	122.0 (3)	H15A—C15—H15C	109.5
N3—C7—C8	116.6 (3)	H15B—C15—H15C	109.5
C9—C8—C13	116.7 (2)		

### Hydrogen-bond geometry (Å, °)

**Table:**

*D*—H···*A*	*D*—H	H···*A*	*D*···*A*	*D*—H···*A*
N3—H3···O1^i^	0.89 (1)	2.16 (1)	3.035 (3)	166 (3)

Symmetry codes: (i) −*x*+3/2, *y*−1/2, *z*.
